# Elastic and dynamical structural properties of La and Mn-doped SrTiO_3_ studied by neutron scattering and their relation with thermal conductivities

**DOI:** 10.1038/s41598-018-27984-z

**Published:** 2018-06-25

**Authors:** Ryoichi Kajimoto, Mitsutaka Nakamura, Naoki Murai, Shin-ichi Shamoto, Takashi Honda, Kazutaka Ikeda, Toshiya Otomo, Hiroto Hata, Takahiro Eto, Masaaki Noda, Hideki Kuwahara, Tetsuji Okuda

**Affiliations:** 1 Materials and Life Science Division, J-PARC Center, Tokai, Ibaraki, 319-1195 Japan; 2 Advanced Science Research Center, Japan Atomic Energy Agency, Tokai, Ibaraki, 319-1195 Japan; 3 Institute of Materials Structure Science, High Energy Accelerator Research Organization, Oho, Ibaraki, 305-0801 Japan; 4 Graduate School of Science and Engineering, Kagoshima University, Kagoshima, 890-0065 Japan; 5 Department of Physics, Sophia University, Chiyoda, Tokyo, 102-8554 Japan

## Abstract

The electron-doped SrTiO_3_ exhibits good thermoelectric properties, which makes this material a promising candidate of an n-type oxide thermoelectric device. Recent studies indicated that only a few percent co-doping of La and Mn in SrTiO_3_ substantially reduces the thermal conductivity, thereby greatly improving the thermoelectric figure of merit at room temperature. Our time-of-flight neutron scattering studies revealed that by doping both La and Mn into SrTiO_3_, the inelastic scattering spectrum shows a momentum-independent increase in the low-energy spectral weight approximately below 10 meV. The increase in the low-energy spectral weight exhibits a clear correlation with thermal conductivity. The correlation is attributed to dynamical and local structural fluctuations caused by the Jahn-Teller instability in Mn^3+^ ions coupled with the incipient ferroelectric nature of SrTiO_3_, as the origin of the low thermal conductivity.

## Introduction

Thermoelectric device is one of the key devices for the sustainable energy technology. It was found that electron-doped SrTiO_3_ exhibits a high thermoelectric power factor at room temperature (RT) comparable to that of a prototypical thermoelectric semiconductor Bi_2_Te_3_^1^, which makes this material a promising candidate of an n-type oxide thermoelectric material and a counterpart of a p-type Co oxide thermoelectric material^2^. The high thermopower of SrTiO_3_ was attributed to its heavy effective mass and incipient ferroelectrics^1,3^. The thermoelectric performance is expressed as the dimensionless figure of merit *ZT* = *S*^2^*σT*/*κ*, where *S*, *σ*, *T*, and *κ* denote the Seebeck coefficient, electric conductivity, temperature, and thermal conductivity, respectively. If *ZT* was further improved, an environmental benign thermoelectric device composed of non-toxic, non-volatile, and abundant elements could be realised. In order to achieve a higher *ZT* around RT, it is necessary to suppress *κ* while maintaining high value of *σ*. The electronic contribution of the thermal conductivity *κ*_e_ in the electron-doped SrTiO_3_ as estimated by the Wiedemann-Frantz law is less than 10^−1^*κ*_l_^4^. Therefore, suppression of the phonon contribution *κ*_l_ is the key. A conventional approach for this purpose involves introducing some local disorders, such as defects or nanostructures, to increase phonon scattering. However, static disorders also decrease *σ* severely such that *ZT* does not improve^4,5^.

Recently, we found that a few percent co-doping of La and Mn in SrTiO_3_ substantially reduces the thermal conductivity at room temperature without significant reduction of the electric conductivity^6,7^. Interestingly, this type of anomaly occurs only when both of La and Mn are doped, and it potentially corresponds to a dynamical effect coupled with carriers. The substitution of Sr^2+^ ions by La^3+^ ions results in the electron doping. Further doping of the Mn ions are expected to create Jahn-Teller active Mn^3+^ ions. SrTiO_3_ is a well-known incipient ferroelectric material that is characterised by a soft TO phonon at the Γ point^8,9^. Additionally, it exhibits a cubic to tetragonal structure transition at *T*_s_ = 105 K, and this is caused by antiferrodistortive distortions of TiO_6_ octahedra characterised by the softening of a phonon mode at the R point^10^. The local Jahn-Teller distortions induced by the co-doping can work as strong phonon scatterers, and the effect is enhanced if they coupled with the inherent structural instability of SrTiO_3_. This expectation reminds us the idea for skutterdites and clathrates that local motions of atoms in cages termed as rattling intervene in the heat flows without significantly affecting the electron conductions^11–13^, although later it was revealed that the rattling modes are rather low-lying optical modes with weak interactions with the host lattices^14–16^. It is interesting to consider whether the doped SrTiO_3_ exhibits similarities to skutterdites and clathrates. The fore-mentioned findings and expectations motivated us to investigate the structural properties of La and Mn-doped SrTiO_3_. Here, we performed time-of-flight elastic and inelastic neutron scattering measurements on powder samples of La and Mn-doped SrTiO_3_, Sr_0.95_La_0.05_TiO_3_ (SLTO), SrTi_0.98_Mn_0.02_O_3_ (STMO), Sr_0.95_La_0.05_Ti_0.98_Mn_0.02_O_3_ (SLTMO2), and Sr_0.95_La_0.05_Ti_0.96_Mn_0.04_O_3_ (SLTMO4), and non-doped SrTiO_3_ (STO) to examine the doping dependence of the static as well as dynamical structural properties.

## Results and Discussion

Okuda *et al*.^7^ reported that at 300 K, STO, STMO, and SLTO exhibit similar values of *κ* while SLTMO2 exhibits significantly lower *κ*, and the latter is approximately half of that for the former three samples. We studied *κ* in the range of 0 ≤ *x* ≤ 0.07 and 0 ≤ *y* ≤ 0.04 in Sr_1−*x*_La_*x*_Ti_1−*y*_Mn_*y*_O_3_, and found that the sample with *x* = 0.05 and *y* = 0.02 (SLTMO2) gives the lowest *κ*. Therefore, further doping of Mn compared with SLTMO2 does not cause further decrease in *κ*, and *κ* for SLTMO4 exceeds that for SLTMO2, although it is still substantially lower than *κ*'s for STO, STMO, and SLTO. The phonon thermal conductivity *κ* is expressed as ${\kappa }_{{\rm{l}}}=\frac{1}{3}C{v}_{{\rm{g}}}l$, where *C*, *v*_g_, and *l* denote the specific heat, group velocity, and mean free path of phonons, respectively. We found all the compounds exhibit similar values of specific heats at high temperatures (see Supplementary Fig. S1) although doping dependence was reported at low temperatures below 100 K^7,17^. This result indicates that the large reduction of *κ* in SLTMO2 and SLTMO4 is not caused by the change in *C* and instead is caused by the decrease in *l* or *v*_g_. In particular, the reduction of *l* is related to the static or dynamical disorder of structures, and we expect to detect it through the neutron scattering measurements.

**Figure 1:**
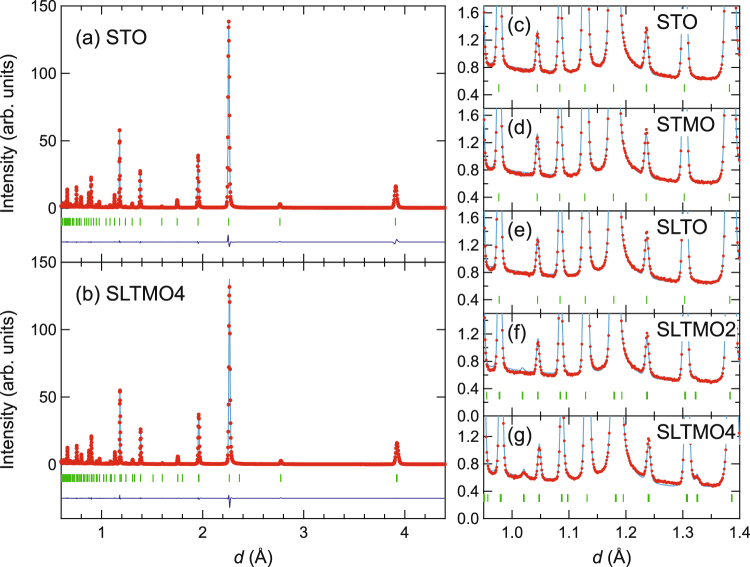
Powder diffraction patterns and their Rietveld refinements for the five samples in the study. The Rietveld refinements for STO, STMO, and SLTO were performed with the cubic *Pm*$\bar{3}$*m* space group, while those for SLTMO2 and SLTMO4 were performed with the tetragonal *I*4/*mcm*. Red symbols and light blue lines denote the observed and calculated diffraction patterns, respectively. Dark blue lines in (**a**,**b**) denote the difference between the observed and calculated diffraction patterns. Green vertical bars denote calculated peak positions. (**a**,**b**) The full diffraction patterns of (**a**) STO and (**b**) SLTMO4. (**c**–**g**) Parts of the diffraction patterns in the region of *d* = 0.95–1.4 Å for (**c**) STO, (**d**) STMO, (**e**) SLTO, (**f**) SLTMO2, and (**g**) SLTMO4.

Figure 1(a,b) shows powder neutron diffraction patterns of STO and SLTMO4, respectively, as functions of *d*-spacings. Figure 1(c–g) shows expansions of the powder patterns for the five samples in the region of *d* = 0.95–1.4 Å. All the powder diffraction patterns were refined with a single phase by the Rietveld refinements, and did not exhibit observable peak broadening, thereby indicating that the cation doping did not induce considerable impurity phases or disorder. The structural parameters obtained by the Rietveld refinements are compiled in Supplementary Tables S1 and S2. The diffraction pattern of STO was successfully fitted to the cubic structure with the space group $Pm\bar{3}m$. Conversely, the diffraction pattern of SLTMO4 was fitted to the tetragonal structure with the space group *I*4/*mcm*, and this is the same structure as the low-temperature tetragonal phase of SrTiO_3_^18–20^. The tetragonal structure is characterised by superlattice peaks with indices $\frac{h}{2}\frac{k}{2}\frac{l}{2}$ in the cubic setting that were observed at, e.g., *d* = 1.02 Å and 1.325 Å in Fig. 1(g). The superlattice peaks were not observed in the powder diffraction patterns of STMO and SLTO [Fig. 1(d,e)], and they were also adequately described by the cubic structural model with *R* factors similar to STO (Supplementary Table S1). On the other hand, in SLTMO2, the refinement with the cubic structure results in a considerably higher *R* factor (*R*_wp_ = 4.5%) when compared with those for the other compounds (Supplementary Table S1). Although we did not observe any peak splitting or broadening, we observed tiny shoulders at the tetragonal superlattice peak positions [for example, at *d* = 1.325 Å in Fig. 1(f)]. Then, we refined the diffraction pattern with the tetragonal structure, and found it yields a reasonably low *R* factor (Supplementary Table S2). Therefore, the crystal structure of SLTMO2 is most likely tetragonal, although its tetragonal distortion is small.

The structural change from cubic to tetragonal by the La and Mn co-doping is qualitatively explained in terms of the tolerance factor $t=({r}_{{\rm{A}}}+{r}_{{\rm{O}}})/\sqrt{2}({r}_{{\rm{B}}}+{r}_{{\rm{O}}})$, where *r*_A_, *r*_B_, and *r*_O_ denote the ionic radii of the A and B sites, and oxygen ion, respectively, in the ABO_3_ perovskite structure. SrTiO_3_ is in a critical state with *t* = 1.002 based on the Shannon radii^21^. Zhong and Vanderbilt showed by first-principles calculations that lower *t* enhances the antiferrodistortive structural instability and leads to a tetragonal structural transition^22^. The ionic radius of La^3+^ is smaller than that of Sr^2+^, and that of Mn^3+^ is larger than that of Ti^4+^. Therefore, the co-doping of La and Mn is expected to result in a decrease in *t*. This is consistent with the tendency to stabilise the tetragonal structure. However, we note that the change in the tolerance factor by the La and Mn doping is very small: *t* is 0.999 even in SLTMO4. The high sensitivity of the structural transition on the tolerance factor should reflect that SrTiO_3_ is in a structurally critical state. However, it is unlikely that the tetragonal structure in the La-Mn co-doped samples is the origin of the low *κ*, since the transition in STO to the low-temperature tetragonal structure at *T*_s_ does not significantly change *κ*^7^.

**Figure 2:**
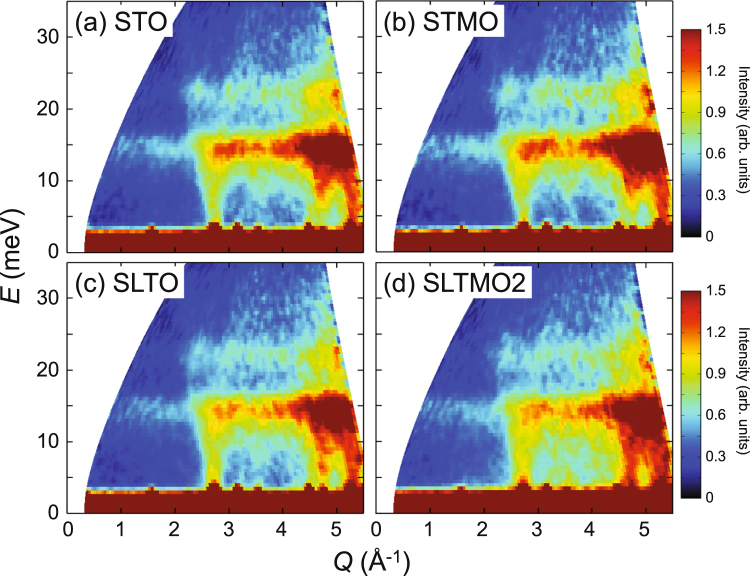
*Q*-*E* maps of the inelastic neutron scattering intensities *S*(*Q*, *E*) for (**a**) STO, (**b**) STMO, (**c**) SLTO, and (**d**) SLTMO2 measured with the incident energy *E*_i_ = 50 meV.

Figure 2 shows the inelastic neutron scattering spectra *S*(*Q*, *E*) for STO, STMO, SLTO, and SLTMO2 represented as intensity maps on the *Q*-*E* planes, where *Q* and *E* denote the momentum and energy transfers, respectively. With respect to STO [Fig. 2(a)], optical modes and zone boundary modes that have flat dispersions^8,9^ are prominently observed at *E* ~ 15 meV and 23 meV by powder-averaging. Additionally, several dispersive excitations are observed. The excitations extending from *E* = 0 meV at *Q* ~ 2.8 Å^−1^ and 5.3 Å^−1^ are attributed to the acoustic or soft TO phonons, since strong 111 and 311 Bragg peaks exist at the *Q* positions. Another dispersive excitation exists with an energy gap from *E* = 0 meV at *Q* ~ 4.8 Å^−1^. The *Q* position corresponds to ${\bf{Q}}=(\frac{5}{2},\frac{3}{2},\frac{1}{2})$, and thus the dispersive excitation is attributed to soft phonons at the R points^9^. The excitation spectra for STMO and SLTO are very similar to that for STO [Fig. 2(b,c)]. In contrast, with respect to SLTMO2, although the overall structure of the spectrum is similar to the other three compounds, the low-energy part approximately below *E* = 15 meV clearly exhibits higher intensity [Fig. 2(d)].

**Figure 3:**
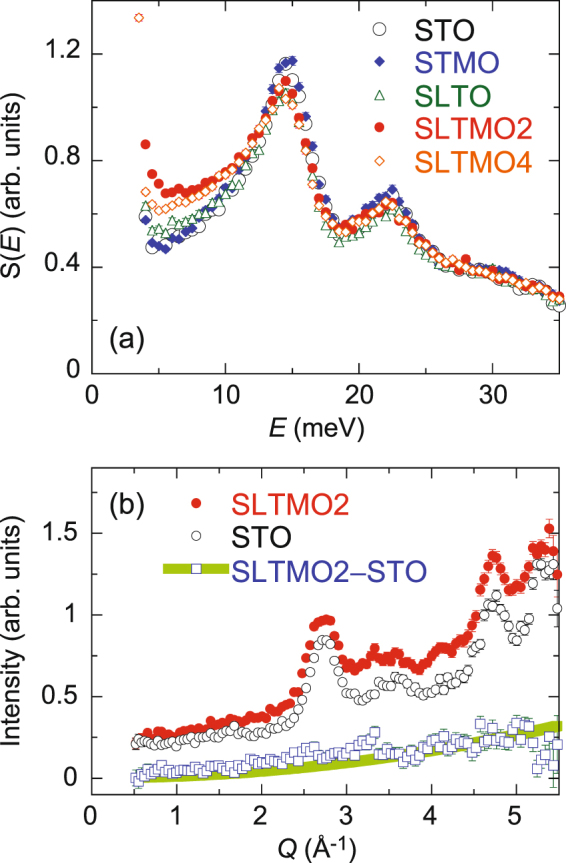
(**a**) *Q*-integrated scattering intensities *S*(*E*) obtained by integrating *S*(*Q*, *E*)’s in Fig. 2 over Q = −0.5–5.5 Å^−1^. Open circles, solid diamonds, open triangles, solid circles, and open diamonds denote *S*(*E*) for STO, STMO, SLTO, SLTMO2, and SLTMO4, respectively. (**b**) Constant-*E* cuts of *S*(*Q*, *E*) for STO (open circles) and SLTMO2 (closed circles) at *E* = 5–10 meV. The open squares denote the difference between the two data. The solid line indicates a fit of the difference intensity to *Q*^2^.

**Figure 4:**
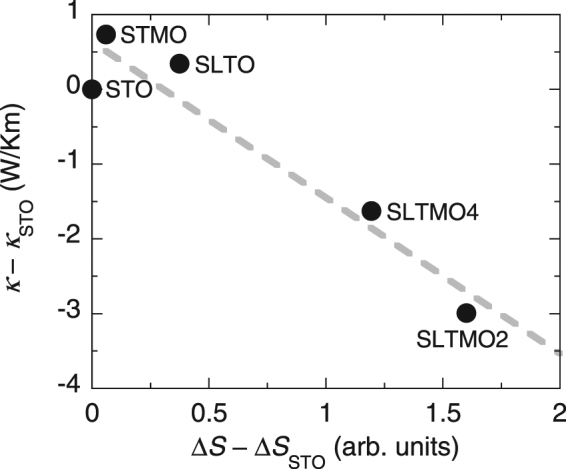
*S*(*E*) integrated over *E* = 5–10 meV relative to thermal conductivities *κ* at 298 K with respect to the values for STO. The values of *κ* are obtained from ref.^7^. The broken line denotes a visual guide.

In order to quantify the increase in the spectral weight in the low-energy region for SLTMO2, we integrated the *S*(*Q*, *E*) over *Q* to obtain the energy dependent spectra *S*(*E*). Figure 3(a) shows *S*(*E*) for all the five samples obtained by integrating their *S*(*Q*, *E*) over *Q* = 0.5–5.5 Å^−1^. The La-Mn co-doped samples exhibit a substantial increase in the spectral weight at $E\lesssim 10$ meV. We note that the increase in the low-energy spectral weight does not simply follow the increase in the Mn content, and SLTMO4 exhibits an even smaller increase than that for SLTMO2. These findings are compared with the fact that the La-Mn co-doped compounds exhibit lower *κ* when compared with the mother compound as well as the Mn or La doped compounds. Subsequently, we compared the values of *S*(*E*) in the low-energy region with the values of *κ*. Figure 4 shows ${\rm{\Delta }}S=\int S(E)\,dE$ in the region of *E* = 5–10 meV relative to the values of *κ* at 298 K^7^. The values for STO are subtracted from these values. Interestingly, Fig. 4 shows a strong correlation between the increase in the low-energy spectral weight and the decrease in *κ*. As we mention in the *Methods* section, there is a 10% ambiguity in the normalisation factors depending on the deriving methods, and this may lead to an ambiguity in the relation between the spectral weight and *κ*. However, we confirmed that the overall trends between *κ* − *κ*_STO_ and Δ*S* − Δ*S*_STO_ in Fig. 4 does not change, although the values of Δ*S* − Δ*S*_STO_ depend on the normalisation factors. The result indicates that the increase in the low-energy spectral weight and the decrease in *κ* come from the same origin.

Given that the tetragonal structure of SrTiO_3_ is induced by the softening of the zone boundary phonons at the R points^10^, it is expected that the increase in the low-energy spectral weight is related to the tetragonal structure. Specifically, an energy gap of the dispersive excitation is observed at *Q* = 4.8 Å^−1^ in the cubic STO [Fig. 2(a)], which is attributed to the soft phonon at ${\bf{Q}}=(\frac{5}{2},\frac{3}{2},\frac{1}{2})$, and it disappears in the tetragonal SLTMO2 [Fig. 2(d)]. In Fig. 3(a), a slight decrease in the spectral weight around the *E* = 15 meV peak in SLTMO2 and SLTMO4 can originate from the softening. However, SLTMO4 exhibits a smaller increase in the low-energy spectral weight when compared with SLTMO2, although it exhibits larger tetragonal structural distortions. Figure 3(b) shows the *Q* dependences of the inelastic scattering spectra for STO and SLTMO2 obtained by integrating their *S*(*Q*, *E*)’s in the range of *E* = 5–10 meV (open circles and closed circles, respectively). The intensity for SLTMO2 exceeds that of STO due to the enhancement in the low-energy spectral weight throughout the *Q* region. The difference between the intensities for both samples does not indicate significant structures (open squares). It increases as *Q* increases with a *Q*^2^ dependence, which evidences that it possesses a lattice origin. These observations strongly suggest that the increase in the low-energy spectral weight is not fully attributed to the tetragonal structural transition, but it is mainly attributed to some local structural fluctuations over such a high energy as ~10 meV. The enhanced low-energy spectra appear to be quasielastic scatterings centred on *E* = 0. On the other hand, they also likely indicate damped optical phonons in the whole *Q* range, considering that the Bose population factor increases with decreasing energy transfer. It is difficult to distinguish which is the case from the present study, and a study with single crystals should be required. These dynamical structural fluctuations are expected to scatter significantly more phonons compared with static local distortions, and thereby effectively decrease the thermal conductivity.

It is surprising that such small amounts of doping of La and Mn induce an extremely clear anomaly as observed by powder inelastic neutron scattering. We consider two possible origins that cause high sensitivity relative to the dopants. One is the fact that the co-doping induces Jahn-Teller active Mn^3+^ ions with the electronic configuration of ${t}_{2g}^{3}{e}_{g}^{1}$. Thus, the local lattice distortions around the Mn ions are enhanced by the Jahn-Teller effect. It is interesting to note that the energy scale of the low-energy scatterings observed in the present study is similar to that for the quasielastic scatterings observed in colossal-magnetoresistive manganites, and these were attributed to the dynamics of the Jahn-Teller polarons^23–25^. The other origin is that SrTiO_3_ is an incipient ferroelectric and exhibits instability to ferroelectric distortions^26–32^. The latter allows local ferroelectric distortions due to a small amount of structural perturbation. Both the scenarios are not necessarily exclusive. It was reported that ferroelectricity is induced in SrTiO_3_ by applying external pressure or strain^26–29^. The local Jahn-Teller distortions can couple with local ferroelectric distortions if the former acts as chemical pressure.

It would be interesting to discuss the relation between the present results and the electronic structure of Sr_1−*x*_La_*x*_Ti_1−*y*_Mn_*y*_O_3_. As mentioned in the introduction, the heavy electron mass is regarded as an important ingredient of the high thermopower in SrTiO_3_^1,3^. A low-temperature specific heat study revealed that the effective mass is increased by the Mn doping in Sr_1−*x*_La_*x*_TiO_3_^7^. The observed local structural fluctuations may also contribute to the renormalisation of the effective mass of the electron through the small-polaron-like electron-lattice coupling.

**Figure 5:**
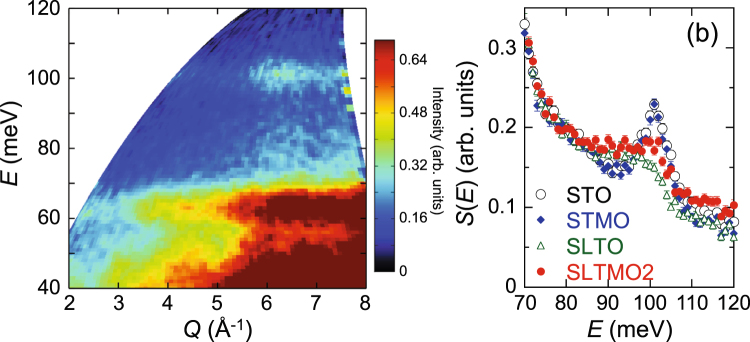
(**a**) *Q*-*E* map of the inelastic neutron scattering intensity *S*(*Q*, *E*) for STO measured with the incident energy *E*_i_ = 131 meV. (**b**) *Q*-integrated scattering intensities *S*(*E*) obtained by integrating *S*(*Q*, *E*)’s in the region of *Q* = 4–7.4 Å^−1^ for STO (open circles), STMO (solid diamonds), SLTO (open triangles), and SLTMO2 (solid circles).

The Jahn-Teller scenario requires creation of Mn^3+^ ions, and this was confirmed by the lattice parameters^6,7^ and specific heat^17^. The creation of Mn^3+^ ions is also supported by the present inelastic neutron scattering spectra at high energies as explained below. Figure 5(a) shows a *Q*-*E* map of a high-energy part of *S*(*Q*, *E*) for STO. The phonon spectrum in this high-energy region is dominated by the contribution from vibrations of the light oxygen ions^33^. It exhibits a large phonon band gap in the range of *E* = 70–90 meV, and this is consistent with a previous study^33^. The highest-energy excitation observed at ~100 meV is dominated by the oxygen vibrations^33^. We performed a phonon mode calculation, and confirmed that it is attributed to the bond-stretching mode related to the deformation of the TiO_6_ octahedra. Figure 5(b) shows the *Q*-integrated phonon spectra of the breathing mode for STO, STMO, SLTO, and SLTMO2, obtained by integrating their *S*(*Q*, *E*) over *Q* = 4.0–7.4 Å^−1^. The spectra of STO and STMO are almost identical and exhibit sharp peaks at *E* ~ 100 meV. Conversely, in SLTO, this peak exhibits a significant decrease in height and broadening in width with a shift of weight to lower energy. The peak height and the peak centre of the 100 meV peak are recovered in SLTMO2. The softening of the bond-stretching mode toward the zone boundary along with carrier doping was commonly observed in cubic and layered perovskite oxides^34^. The anomalies in the 100 meV peak in SLTO are interpreted as similar softening of the bond-stretching mode caused by carrier doping. Consequently, the recovery of the bond-stretching mode in SLTMO2 is most likely caused by the decrease in electron concentration due to the creation of Mn^3+^ ions.

## Conclusion

In this study, we examined elastic and dynamical structural properties in La and Mn-doped SrTiO_3_ by neutron scattering. Co-doping of La and Mn causes a tetragonal structure despite tiny changes in the tolerance factor, which reflects the structurally critical nature of SrTiO_3_. The inelastic scattering measurements revealed that a momentum-independent increase in the low-energy spectral weight approximately below 10 meV in La and Mn co-doped SrTiO_3_. It is not solely explained by the tetragonal structural transition, and instead is attributable to some local structural fluctuations. Furthermore, we found the increase in the low-energy spectral weight exhibits a clear correlation with thermal conductivity. We consider that dynamical and local structural fluctuations caused by the Jahn-Teller instability in Mn^3+^ ions coupled with the incipient ferroelectric nature of SrTiO_3_ induce additional low-energy scatterings and correspond to the origin of low thermal conductivity in the La and Mn co-doped compounds.

## Methods

Powder samples of SrTiO_3_, SrTi_0.98_Mn_0.02_O_3_, Sr_0.95_La_0.05_TiO_3_, Sr_0.95_La_0.05_Ti_0.98_Mn_0.02_O_3_, and Sr_0.95_La_0.05_Ti_0.96_Mn_0.04_O_3_ were prepared by crushing single crystals grown by the floating-zone method. Each of the samples has a weight of approximately 5 g. Although deficiencies of Mn ions were reported for Mn-doped samples^7^, we denote the samples by nominal compositions throughout the study since the amounts of deficiencies are excessively low for detection through neutron diffraction measurements. The specific heats were measured by the relaxation method by using single crystal samples. The powder neutron scattering measurements were performed at room temperature by using the NOVA diffractometer in the Materials and Life Science Experimental Facility (MLF) of the Japan Proton Accelerator Research Complex (J-PARC). Rietveld refinements were performed by using Z-Rietveld (version 1.0.2)^35,36^. The inelastic neutron scattering measurements were performed by using the 4SEASONS direct-geometry spectrometer in MLF^37^. The incident energies of *E*_i_ = 50 meV and 131 meV were simultaneously utilised by using the Fermi chopper rotating at 250 Hz through the multi-*E*_i_ technique^38,39^. Scattering from aluminium constituting the sample environment device was eliminated by using the oscillating radial collimator^40^. The inelastic neutron scattering data were converted to a powder-averaged dynamical scattering function *S*(*Q*, *E*) by using the Utsusemi software package^41^. The difference in utilised neutron flux between data was normalised based on the counts of the proton beam injected into the neutron target. The difference in volume between the samples was normalised such that their inelastic scattering intensities in the region of *E* = 25–35 meV in the *E*_i_ = 50 meV data coincide. The obtained normalisation factors agree with those obtained from the molar numbers or the Bragg peak intensities of the samples within a difference of 10%. The phonon-mode calculation was performed using the density-functional perturbation theory (DFPT)^42^ as implemented in the Quantum ESPRESSO package^43^.

## Electronic supplementary material

Supplementary Information

## References

[1] Okuda T, Nakanishi K, Miyasaka S, Tokura Y (2001). Large thermoelectric response of metallic perovskites: Sr_1−*x*_La_*x*_TiO_3_ (0 ≤ *x* ≤ 0.1). Phys. Rev. B.

[2] Terasaki I, Sasago Y, Uchinokura K (1997). Large thermoelectric power in NaCo_2_O_4_ single crystals. Phys. Rev. B.

[3] Shirai K, Yamanaka K (2013). Mechanism behind the high thermoelectric power factor of SrTiO_3_ by calculating the transport coefficients. J. Appl. Phys..

[4] Fukuyado J, Narikiyo K, Akaki M, Kuwahara H, Okuda T (2012). Thermoelectric properties of the electrondoped perovskites Sr_1−*x*_Ca_*x*_Ti_1−*y*_Nb_*y*_O_3_. Phys. Rev. B.

[5] Buscaglia MT (2014). Effect of nanostructure on the thermal conductivity of La-doped SrTiO_3_ ceramics. J. Eur. Ceram. Soc..

[6] Okuda T (2014). Effects of Mn substitution on the thermoelectric properties of the electron-doped perovskite Sr_1−*x*_La_*x*_TiO_3_. J. Phys.: Conf. Ser..

[7] Okuda T (2016). Effects of Mn substitution on the thermoelectric properties and thermal excitations of the electron-doped perovskite Sr_1−*x*_La_*x*_TiO_3_. J. Phys. Soc. Jpn..

[8] Cowley RA (1964). Lattice dynamics and phase transitions of strontium titanate. Phys. Rev..

[9] Stirling WG (1972). Neutron inelastic scattering study of the lattice dynamics of strontium titanate: harmonic models. J. Phys. C: Solid State Phys.

[10] Shirane G, Yamada Y (1969). Lattice-dynamical study of the 110 °K phase transition in SrTiO_3_. Phys. Rev..

[11] Keppens V (1998). Localized vibrational modes in metallic solids. Nature.

[12] Hermann RP (2003). Einstein oscillators in thallium filled antimony skutterudites. Phys. Rev. Lett..

[13] Christensen M, Juranyi F, Iversen BB (2006). The rattler effect in thermoelectric clathrates studied by inelastic neutron scattering. Physica B.

[14] Lee CH, Hase I, Sugawara H, Yoshizawa H, Sato H (2006). Low-lying optical phonon modes in the filled skutterudite CeRu_4_Sb_12_. J. Phys. Soc. Jpn..

[15] Koza M (2008). Breakdown of phonon glass paradigm in La- and Ce-filled Fe_4_Sb_12_ skutterudites. Nat. Mater..

[16] Christensen M (2008). Avoided crossing of rattler modes in thermoelectric materials. Nat. Mater..

[17] Okuda T (2017). Low temperature specific heat in lightly Mn-substituted electron-doped SrTiO_3_. J. Phys. Soc. Jpn..

[18] Unoki H, Sakudo T (1967). Electron spin resonance of Fe^3+^ in SrTiO_3_ with special reference to the 110 °K phase transition. J. Phys. Soc. Jpn..

[19] Fujishita H, Shiozaki Y, Sawaguchi E (1979). X-ray crystal structure analysis of low temperature phase of SrTiO_3_. J. Phys. Soc. Jpn..

[20] Kiat JM, Roisnel T (1996). Rietveld analysis of strontium titanate in the Müller state. J. Phys.: Condens. Matter.

[21] Shannon RD (1976). Revised effective ionic radii and systematic studies of interatomic distances in halides and chalcogenides. Acta Crystallogr., Sect. A.

[22] Zhong W, Vanderbilt D (1995). Competing structural instabilities in cubic perovskites. Phys. Rev. Lett..

[23] Argyriou DN (2002). Glass transition in the polaron dynamics of colossal magnetoresistive manganites. Phys. Rev. Lett..

[24] Lynn JW (2007). Order and dynamics of intrinsic nanoscale inhomogeneities in manganites. Phys. Rev. B.

[25] Chen Y (2008). Polaron formation in the optimally doped ferromagnetic manganites La_0.7_Sr_0.3_MnO_3_ and La_0.7_Ba_0.3_MnO_3_. Phys. Rev. B.

[26] Burke W, Pressley R (1971). Stress induced ferroelectricity in SrTiO_3_. Solid State Commun.

[27] Uwe H, Sakudo T (1976). Stress-induced ferroelectricity and soft phonon modes in SrTiO_3_. Phys. Rev. B.

[28] Tikhomirov O, Jiang H, Levy J (2002). Local ferroelectricity in SrTiO_3_ thin films. Phys. Rev. Lett..

[29] Haeni JH (2004). Room-temperature ferroelectricity in strained SrTiO_3_. Nature.

[30] Bednorz JG, Müller KA (1984). Sr_1−*x*_Ca_*x*_TiO_3_: An *XY* quantum ferroelectric with transition to randomness. Phys. Rev. Lett..

[31] Hemberger J, Lunkenheimer P, Viana R, Böhmer R, Loidl A (1995). Electric-field-dependent dielectric constant and nonlinear susceptibility in SrTiO_3_. Phys. Rev. B.

[32] Itoh M (1999). Ferroelectricity induced by oxygen isotope exchange in strontium titanate perovskite. Phys. Rev. Lett..

[33] Choudhury N, Walter EJ, Kolesnikov AI, Loong C-K (2008). Large phonon band gap in SrTiO_3_ and the vibrational signatures of ferroelectricity in *A*TiO_3_ perovskites: First-principles lattice dynamics and inelastic neutron scattering. Phys. Rev. B.

[34] Braden M, Reichardt W, Shiryaev S, Barilo S (2002). Giant phonon anomalies in the bond-stretching modes in doped BaBiO_3_: comparison to cuprates manganites and nickelates. Physica C.

[35] Oishi R (2009). Rietveld analysis software for J-PARC. Nucl. Instrum. Methods Phys. Res., Sect. A.

[36] Oishi-Tomiyasu R (2012). Application of matrix decomposition algorithms for singular matrices to the Pawley method in *Z*-*Rietveld*. J. Appl. Crystallogr..

[37] Kajimoto R (2011). The Fermi chopper spectrometer 4SEASONS at J-PARC. J. Phys. Soc. Jpn..

[38] Nakamura M (2009). First demonstration of novel method for inelastic neutron scattering measurement utilizing multiple incident energies. J. Phys. Soc. Jpn..

[39] Russina M, Mezei F (2009). First implementation of repetition rate multiplication in neutron spectroscopy. Nucl. Instrum. Methods Phys. Res., Sect. A.

[40] Nakamura M (2015). Oscillating radial collimators for the chopper spectrometers at MLF in J-PARC. JPS Conf. Proc..

[41] Inamura Y, Nakatani T, Suzuki J, Otomo T (2013). Development status of software “Utsusemi” for chopper spectrometers at MLF, J-PARC. J. Phys. Soc. Jpn..

[42] Baroni S, de Gironcoli S, Dal Corso A, Giannozzi P (2001). Phonons and related crystal properties from densityfunctional perturbation theory. Rev. Mod. Phys..

[43] Giannozzi P (2009). QUANTUM ESPRESSO: a modular and open-source software project for quantum simulations of materials. J. Phys.: Condens. Matter.

