# Supplementary material for: Elastic and dynamical structural properties of La and Mn-doped SrTiO3 studied by neutron scattering and their relation with thermal conductivities
Source: Sci Rep. 2018 Jun 25;8:9651. doi: 10.1038/s41598-018-27984-z (PMC6018226; doi:10.1038/s41598-018-27984-z)
Supplement: Supplementary file 1 — Supplementary Information [file 41598_2018_27984_MOESM1_ESM.pdf]

Supplementary Information: Elastic and dynamical structural properties of La and Mn-doped $SrTiO_3$ studied by neutron scattering and their relation with thermal conductivities

Ryoichi Kajimoto[1,*], Mitsutaka Nakamura[1], Naoki Murai[1], Shin-ichi Shamoto[2], Takashi Honda[3,1], Kazutaka Ikeda[3,1], Toshiya Otomo[3,1], Hiroto Hata[4], Takahiro Eto[4], Masaaki Noda[5], Hideki Kuwahara[5], and Tetsuji Okuda[4]

[1]Materials and Life Science Division, J-PARC Center, Tokai, Ibaraki 319-1195, Japan
[2]Advanced Science Research Center, Japan Atomic Energy Agency, Tokai, Ibaraki 319-1195, Japan
[3]Institute of Materials Structure Science, High Energy Accelerator Research Organization, Oho, Ibaraki 305-0801, Japan
[4]Graduate School of Science and Engineering, Kagoshima University, Kagoshima 890-0065, Japan
[5]Department of Physics, Sophia University, Chiyoda, Tokyo 102-8554, Japan
[*]ryoichi.kajimoto@j-parc.jp

Specific Heats

Figure S1 shows temperature dependences of the specific heats (C) for the doped samples, $SrTi_{0.98}Mn_{0.02}O_3$ (STMO), $Sr_{0.95}La_{0.05}TiO_3$ (SLTO), $Sr_{0.95}La_{0.05}Ti_{0.98}Mn_{0.02}O_3$ (SLTMO2), and $Sr_{0.95}La_{0.05}Ti_{0.96}Mn_{0.04}O_3$ (SLTMO4). They were measured by the relaxation method by using single crystal samples. The literature values for $SrTiO_3$ (STO) in Ref. 1 is also shown.

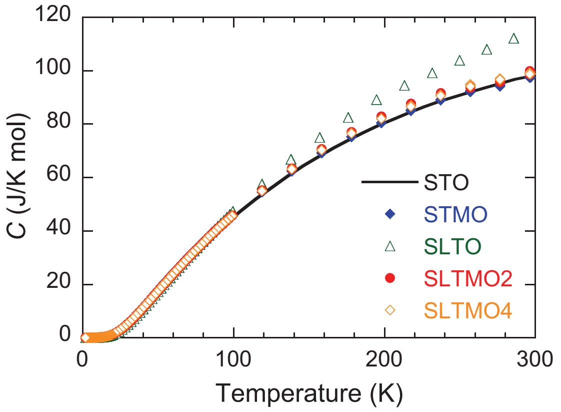

Figure S1. Temperature dependences of specific heats of STO (solid line), STMO (solid diamonds), SLTO (open triangles), SLTMO2 (solid circles), and SLTMO4 (open diamonds). The data of STO is reproduced from Ref. 1.

Crystal Structures

The powder neutron scattering measurements were performed at room temperature by using the NOVA diffractometer in the Materials and Life Science Experimental Facility (MLF) of the Japan Proton Accelerator Research Complex (J-PARC). Rietveld refinements were performed by using Z-RIETVELD (version 1.0.2).[2,3] The powder diffraction patterns of STO, STMO, and SLTO were fitted to the cubic model with the space group $Pm\bar{3}m$, while that of SLTMO4 was fitted to the tetragonal model with the space group $I4/mcm$. The powder diffraction pattern of SLTMO2 was fitted to both the cubic and tetragonal model. Typical observed and calculated diffraction patterns are shown in Fig. 1 in the main text. Tables S1 and S2 compile the structural parameters obtained by the Rietveld refinements for the cubic and tetragonal models, respectively.

		STO	STMO	SLTO	SLTMO2
a (Å)		3.902409(9)	3.901742(2)	3.904083(2)	3.906148(2)
Sr/La	B_{11} (Å^2)	0.545(1)	0.548(1)	0.545(1)	0.506(1)
Ti/Mn	B_{11} (Å^2)	0.234(2)	0.238(2)	0.211(2)	0.180(2)
O	B_{11} (Å^2)	0.777(2)	0.781(2)	0.852(2)	0.853(2)
	B_{33} (Å^2)	0.250(3)	0.263(3)	0.248(3)	0.272(3)
Ti–O (Å)		1.951204(5)	1.950871(1)	1.952042(1)	1.953074(1)
R_{wp} (%)		3.5	3.7	3.7	4.5

Table S1. Crystal structure parameters and Ti–O bond lengths of STO, STMO, SLTO, and SLTMO2 obtained by the Rietveld refinements based on the cubic model. The space group is $Pm\bar{3}m$ with the following atomic positions: Sr/La at 1a $(0,0,0)$, Ti/Mn at 1b $(\frac{1}{2},\frac{1}{2},\frac{1}{2})$, and O at 3c $(\frac{1}{2},\frac{1}{2},0)$.

		SLTMO2	SLTMO4
a (Å)		5.52189(2)	5.53367(2)
c (Å)		7.81745(6)	7.83694(6)
Sr/La	B_{11} (Å^2)	0.779(9)	0.748(8)
	B_{33} (Å^2)	0.03(1)	0.17(1)
Ti/Mn	B_{11} (Å^2)	0.31(2)	0.36(1)
	B_{33} (Å^2)	0.06(3)	0.14(2)
O1	B_{11} (Å^2)	1.18(2)	1.51(2)
	B_{33} (Å^2)	0.30(2)	0.33(2)
O2	x	0.24716(4)	0.24575(2)
	B_{11} (Å^2)	0.529(9)	0.480(6)
	B_{33} (Å^2)	0.62(1)	0.68(1)
	B_{12} (Å^2)	0.30(1)	0.21(1)
Ti–O1 (Å)		1.95438(2)	1.95924(1)
Ti–O2 (Å)		1.952399(9)	1.956729(8)
R_{wp} (%)		3.9	3.5

Table S2. Crystal structure parameters and Ti–O bond lengths of SLTMO2 and SLTMO4 obtained by the Rietveld refinements based on the tetragonal model. The space group is $I4/mcm$ with the following atomic positions: Sr/La at 4b $(0,\frac{1}{2},\frac{1}{4})$, Ti/Mn at 4c $(0,0,0)$, O1 at 4a $(0,0,\frac{1}{4})$, and O2 at 8h $(x,x+\frac{1}{2},0)$.

References

1. Todd, S. S. & Lorenson, R. E. Heat capacities at low temperatures and entropies at 298.16°K. of metatitanates of barium and strontium. *J. Am. Chem. Soc.* **74**, 2043–2045 (1952).

2. Oishi, R. *et al.* Rietveld analysis software for J-PARC. *Nucl. Instruments Methods Phys. Res. Sect. A: Accel. Spectrometers, Detect. Assoc. Equip.* **600**, 94–96 (2009).

3. Oishi-Tomiyasu, R. *et al.* Application of matrix decomposition algorithms for singular matrices to the Pawley method in *Z-Rietveld*. *J. Appl. Crystallogr.* **45**, 299–308 (2012).
